# Home-Helps

**Published:** 1920-01-24

**Authors:** 


### Home-Helps.

A Scheme for Hull.

Though, under the Act, local authorities are empowered to provide "home-helps," few have yet attempted to organise this municipal service. Hull Corporation Health Committee, however, has just made arrangements which should aid other authorities anxious to make a start in the same direction. The medical officer was instructed to advertise for applicants, and those who are accepted as suitable for training are to be referred, first to one of the infant welfare centres for their training, and, secondly, to one of the Education Committee's home-making centres to receive the remainder of their instruction. On passing from the training centres they will be attached to one of the maternity centres and receive their instructions from the medical officer of the centre. Some guarantee is to be obtained binding the women to work for at least six months in the city after their training is completed. The rate of payment is to be on a graduated scale according to the number of children in each house visited, for it would be obviously unfair to fix a flat rate.

The following scale of weekly wages is recommended: In cases where the family consists of one child, £1 per week and meals (or alternatively 2s. 6d. per day for food); two children, £1 1s. 3d.; and three children, £1 2s. 6d.; *plus* 1s. 3d. for each child above this number, making 30s. per week for a family consisting of nine children.

Home-helps are to be supplied free of charge to those who come within the poverty scale of weekly income fixed by the Committee, and those whose incomes exceed that scale are to pay what balance of weekly income remains after applying the scale and allowing deductions for payment of rent and insurance.

Each health visitor is held responsible for the supervision of home helps employed at cases in her particular district. On receiving the application from the Clinical Medical Officer, the health visitor should make a preliminary visit to arrange dates, times, etc., and at least one visit per week, while the "help" is working, to inspect the work.

The Health Committee observes that the number of home-helps is likely to be small, but, provided that a fair number come forward for training, an emergency list will prove very useful to gauge the suitability and capacity of women before they are promoted to the regular list.

Regarding selection, the Committee has come to the conclusion that helps should have good health and be preferably over thirty and under fifty-five years of age. They must be of suitable character, and preference should be given widows and others who are entirely or mainly dependent on their own earnings.

Training includes practical instruction in plain and invalid cookery, food values and prices, laundry work, mending, infant care, and hygiene.

The duties are the ordinary domestic duties usually undertaken by the mother, including cleaning, cooking, working, care of children, mending, and shopping. They are forbidden to undertake the duties of a nurse or midwife or to go to confinement cases unless a nurse or midwife is in attendance; whilst the following rules must be strictly observed:—

(1) They must not refuse to go to the houses where they are sent.

(2) They must be clean, tidy, and punctual. They must wear an apron or overall.

(3) They are forbidden to borrow anything whatever from the houses where they are working.

(4) They are forbidden to receive any money from the person in whose house they are working.

(5) In all matters of health they must act under the orders of the nurse or midwife.

